# Distributed Acoustic Sensing for Monitoring Linear Infrastructures: Current Status and Trends

**DOI:** 10.3390/s22197550

**Published:** 2022-10-05

**Authors:** Hong-Hu Zhu, Wei Liu, Tao Wang, Jing-Wen Su, Bin Shi

**Affiliations:** 1 School of Earth Sciences and Engineering, Nanjing University, Nanjing 210023, China; 2 Nanjing University High-Tech Institute at Suzhou, Suzhou 215123, China; 3 Nanjing Center, China Geological Survey, Nanjing 210016, China

**Keywords:** distributed acoustic sensing (DAS), linear infrastructure, field monitoring, distributed fiber-optic sensing

## Abstract

Linear infrastructures, such as railways, tunnels, and pipelines, play essential roles in economic and social development worldwide. However, under the influence of geohazards, earthquakes, and human activities, linear infrastructures face the potential risk of damage and may not function properly. Current monitoring systems for linear infrastructures are mainly based on non-contact detection (InSAR, UAV, GNSS, etc.) and geotechnical instrumentation (extensometers, inclinometers, tiltmeters, piezometers, etc.) techniques. Regarding monitoring sensitivity, frequency, and coverage, most of these methods have some shortcomings, which make it difficult to perform the accurate, real-time, and comprehensive monitoring of linear infrastructures. Distributed acoustic sensing (DAS) is an emerging sensing technology that has rapidly developed in recent years. Due to its unique advantages in long-distance, high-density, and real-time monitoring, DAS arrays have shown broad application prospects in many fields, such as oil and gas exploration, seismic observation, and subsurface imaging. In the field of linear infrastructure monitoring, DAS has gradually attracted the attention of researchers and practitioners. In this paper, recent research and the development activities of applying DAS to monitor different types of linear infrastructures are critically reviewed. The sensing principles are briefly introduced, as well as the main features. This is followed by a summary of recent case studies and some critical problems associated with the implementation of DAS monitoring systems in the field. Finally, the challenges and future trends of this research area are presented.

## 1. Introduction

Linear infrastructures, such as railways, highways, pipelines, tunnels, embankments and power transmission lines, are a general type of civil infrastructures with long spans. They have the characteristics of long lengths, wide distribution ranges, and long service lives and they are widely distributed in complex and changeable natural environments. They form the backbone of society and play important roles in economic and social development. However, due to the influence of geohazards, earthquakes, corrosion, aging, human activities, and other multiple factors, linear infrastructures face the potential risk of structural deterioration and damage during their service lives [1]. In the past few decades, accidental extraordinary events among linear infrastructures in long-term operations have aroused widespread concern. For instance, water, oil, and gas pipelines have sometimes been damaged by nearby earthworks, landslides, and third-party invasions [2,3]; some dramatic traffic disasters have been caused by rail rust and land subsidence; in some countries, border protection walls and barriers have been damaged by illegal immigration. Therefore, to ensure the safe and reliable operation of linear infrastructures and prevent potential threats in the long run, robust and efficient monitoring systems are required.

The establishment of monitoring and early warning systems for linear infrastructures is key to ensuring their health conditions and reducing the occurrence of disasters. At present, linear engineering monitoring mainly relies on manual inspections and field monitoring. The former is relatively expensive and intermittent [4]. For the latter, the commonly used methods are categorized into two types, i.e., remote sensing and contact monitoring methods. Interferometric synthetic aperture radar (InSAR) and global navigation satellite systems (GNSS) are two popular remote sensing technologies with millimeter-level accuracy for displacement monitoring [5,6]. In recent years, unstaffed aerial vehicle (UAV) photogrammetry and terrestrial laser scanning (TLS) have provided effective solutions for ground surface deformation monitoring with higher accuracy [7]. Although these technologies can obtain large-scale deformation data, monitoring is periodic and only applicable to surface deformations. For subsurface monitoring, a series of geotechnical instruments have been developed, e.g., extensometers, inclinometers, tiltmeters, and piezometers [8]. These can be installed in boreholes of different depths or directly fixed on linear infrastructures to carry out performance monitoring. In this way, the automatic and continuous monitoring of key physical parameters can be enabled, as well as early warnings of abnormal states or accidents. However, most of the instruments are based on single-point measurements and struggle to realize long-distance monitoring. For the above reasons, powerful and robust monitoring technologies for linear infrastructures are urgently needed to provide accurate and comprehensive measurements in real time.

Distributed acoustic sensing (DAS) is a new type of fiber-optic sensing technology that has rapidly developed in recent years. It not only has the advantages of ordinary fiber-optic sensing technologies (e.g., anti-electromagnetic interference, corrosion resistance, slenderness, and flexibility) but it can also measure dynamic strains (e.g., vibrations and sound waves) along fiber paths in a long-distance, fully distributed, and real-time manner [9]. In the past decade, there have been many successful applications of DAS in the field of geophysical detection, such as vertical seismic profile (VSP) acquisition [10,11,12], hydraulic fracturing monitoring [13,14,15], earthquake observation [16,17,18], and structural detection and imaging [19,20,21]. In the fields of acoustics [22,23] and biology [24,25], DAS has also shown its powerfulness. With the rapid development of demodulation techniques, the applications of DAS have gradually extended from land to ocean [26,27,28], glaciers [29,30], and volcanoes [31,32].

**Figure 1:**
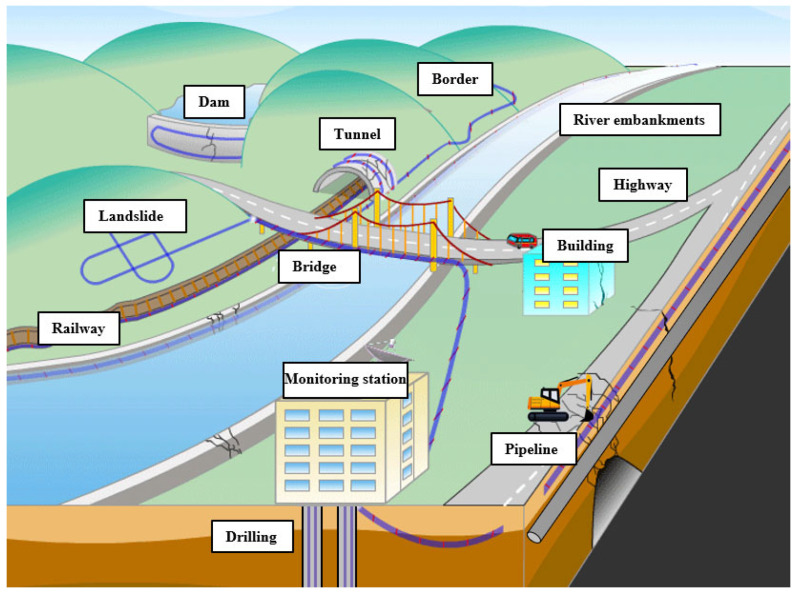
Current and potential application scenarios of DAS for monitoring linear infrastructures and geohazards. Adapted with permission from Ref. [37]. 2017, MDPI publisher.

For linear infrastructures, DAS provides a novel monitoring solution. The large-scale, long-distance, and real-time sensing capabilities of DAS mean that it has unreplaceable advantages for field monitoring. In addition, fiber-optic cables have strong environmental adaptability and can easily collect huge monitoring data along their fiber lengths. Therefore, DAS can be tailored for monitoring linear infrastructures in complex and harsh environments. In recent years, researchers and practitioners worldwide have carried out a large number of field investigations on linear infrastructures using DAS, such as pipeline leakage monitoring [33,34] and rail track health monitoring [35,36]. Figure 1 briefly shows some current and potential application scenarios of DAS for monitoring linear infrastructures and related geohazards.

This paper presents a critical review of the recent developments and applications of DAS for monitoring linear infrastructures. After a brief introduction to the sensing principles, the developmental investigations into applying DAS to monitor different types of linear infrastructures are described in detail. Finally, the major bottlenecks in DAS-based linear infrastructure monitoring are summarized and the development trends of DAS are pointed out. This review is expected to provide valuable insights into the application of DAS for monitoring linear infrastructures and also useful solutions for practitioners and policymakers in related areas.

## 2. Distributed Acoustic Sensing (DAS)

### 2.1. Sensing Principles

**Figure 2:**
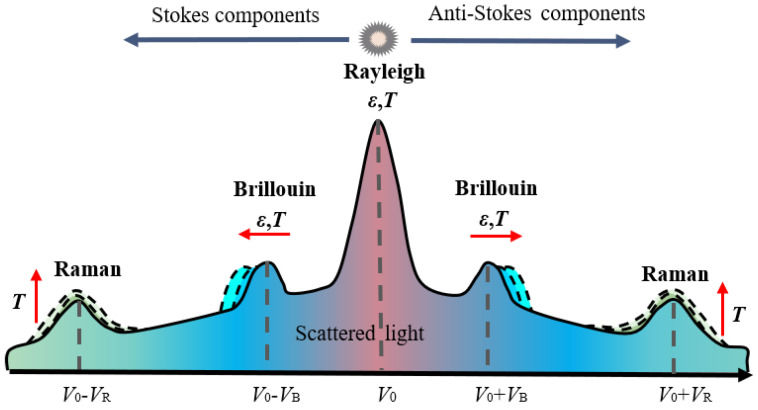
Scattering spectra of an optical fiber. In the figure, *ε* represents strain, *T* represents temperature, *V_0_* is the original light wave frequency, *V_B_* represents the Brillouin shift and *V_R_* represents the Raman shift.

**Figure 3:**
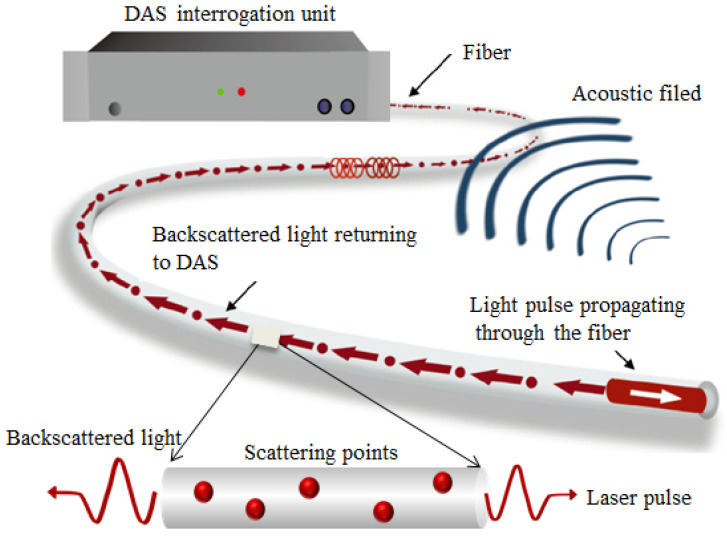
Generic concept of the principles of DAS. Reproduced with permission from Ref. [38]. 2021, John Wiley & Sons.

DAS is a cutting-edge distributed sensing technology that uses light as the information carrier and standard telecommunications-grade optical fibers as the sensing medium for seismic records. Common DAS systems consist of an interrogation unit and a sensing cable. DAS interrogation units continuously inject short-pulse lasers into cables. When light passes through the fiber cores of these cables, the incident light is scattered in different directions due to spatial variations in the refractive index of the fiber cores and different kinds of scattered light are generated, as shown in Figure 2. When optical fibers are disturbed and subjected to strain, temperature, and vibrations, the properties of the scattered light change (wavelength, light intensity, frequency, etc.). By analyzing certain characteristics of scattered light, the changes in various physical parameters (temperature, axial strain, and strain rate) can be revealed. DAS interrogators detect Rayleigh backscattered light along with the fibers and analyze the phase information of coherent Rayleigh scattered light to obtain dynamic strain (vibrations, acoustic waves, etc.) measurements [30], as shown in Figure 3. A review of the literature that introduces the sensing principles of DAS in detail can be found in [16]. As well as Rayleigh backscattered light, Brillouin and Raman scattering light is also generated at every point along the optical fibers. Distributed strain sensing (DSS) and distributed temperature sensing (DTS) can be performed using these scattering phenomena.

### 2.2. Sensing Performance

In the past 10 years, many technological companies and research institutions have carried out research and development for DAS applications. As we all know, the interrogation unit is the sensing “heart” that fundamentally determines the sensing performance (including spatial resolution, detection distance, response capability, etc.). Table 1 briefly shows the basic parameters of several commercially available DAS interrogators.

It can be seen from Table 1 that these interrogation units can meet the needs of large-scale, long-distance, high-density, and real-time detection. They have excellent dynamic response capabilities and can sense a wide band of vibration waves. Therefore, the application of DAS in the field of linear infrastructure monitoring can make full use of its sensing performance and give full play to its sensing advantages.

### 2.3. Installation and Layout of Fiber-Optic Cables

Deformation coupling is one of the key factors affecting the data quality of DAS. When the coupling between a fiber-optic cable and its surroundings is weak, the cable cannot accurately capture external vibration information, which adversely affects the data quality. In recent years, researchers have carried out a large number of experimental studies on the deformation coupling between sensing cables and monitoring objects and have gained abundant knowledge in this aspect [39,40,41,42]. Based on a series of theoretical and experimental studies, Zhu et al. claimed that confining pressure can effectively enhance cable–soil deformation compatibility and expand the deformation sensing range [40]. Zhang et al. proved through a large number of pullout tests that the micro-anchors installed on fiber-optic cables can effectively ensure cable–soil interface bonding [41]. However, for linear infrastructures with large spatial spans, there is still the challenge of guaranteeing that good coupling conditions are always valid during field monitoring.

To enhance the coupling effect between fiber-optic cables and instrumented structures, researchers have designed a variety of cable installation techniques, as listed in Table 2. It is necessary to choose the appropriate cable layout method according to the specific monitoring objects and environments. In a sense, the layout of fiber-optic cables is a key component of DAS monitoring systems, which directly determines the quality of the monitoring data. Therefore, attention must be paid to the optimized installation and layout of cables during field instrumentation.

## 3. Applications of DAS in Linear Infrastructure Monitoring

As a new type of fiber-optic sensing technology, DAS has been widely studied by researchers since its inception. In recent years, practitioners worldwide have tried to apply DAS to monitor a variety of critical linear infrastructures and there have been many successful applications. This section briefly introduces the current applications of DAS in linear infrastructure monitoring and focuses on the conclusions that have been drawn from these case studies.

### 3.1. Railway Safety Monitoring

#### 3.1.1. Train Positioning and Speed Monitoring

Railway management departments worldwide need to accurately monitor train positioning and speed information to ensure the safety of trains. In train positioning and speed monitoring, track circuit technology is widely used [47]. However, in some extreme weather conditions, such as super-strong lightning, track circuit technology may not work properly. Other sensing technologies also have application limitations. For instance, global positioning systems (GPS) have weak sensing capabilities in closed environments, such as tunnels, and cannot obtain accurate train motion information.

**Figure 4:**
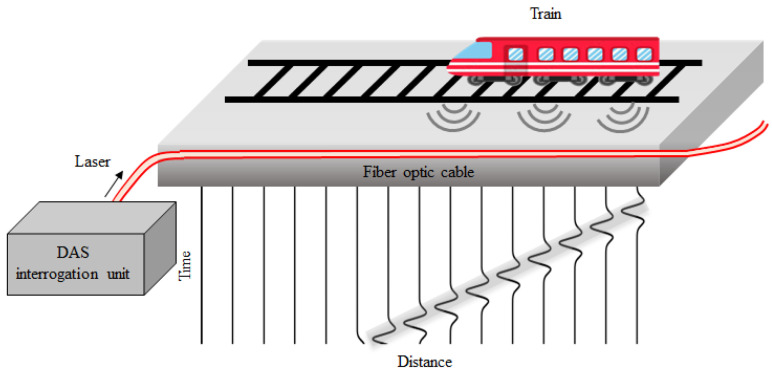
Schematic diagram of train positioning and speed monitoring based on DAS.

The development of DAS has provided a new solution for train positioning and speed monitoring. The advantages of DAS (strong anti-electromagnetic interference, high temporal resolution, etc.) can make up for the shortcomings of existing monitoring technologies. DAS systems are also convenient to use to perform field monitoring by just connecting a sensing cable to an interrogation unit, as shown in Figure 4. Since trains generate strong vibration signals during the running process, sensing cables close to trains vibrate strongly, while sensing cables far away from the trains are undisturbed and only record background noise. Therefore, the position and running speed of trains can be easily determined by analyzing the recorded data from the cables at each position.

As early as 2014, Peng et al. conducted a field study [48]. They laid a 10.2 km sensing cable along a track to record vibration signals as trains were moving. From the DAS data, the position and running speed of trains at various time points could be clearly seen, demonstrating the feasibility of DAS for train positioning and speed monitoring. Since then, an increasing number of field trials have been carried out [49,50,51]. However, utilizing DAS for train positioning and speed monitoring also faces some challenges; for instance, how to bury sensing cables near tracks without affecting railway transportation and how to quickly extract effective information from continuous monitoring data. To process sensing data accurately and quickly, a variety of intelligent algorithms have been developed [36,51,52]. He et al. proposed an improved Canny algorithm for precise train positioning and the feasibility of this algorithm has been verified through field experiments [52]. The results showed that the positioning error is less than 10 m. The same authors also proposed a cubical smoothing algorithm with a five-point approximation to denoise vibration signals and shorten the calculation time. Wiesmeyr et al. proposed a real-time train tracking algorithm that runs on the basis of 1 s signals without delay [36]. This algorithm combines machine learning techniques, such as principal component analysis (PCA) and support vector machines (SVMs), with image processing methods, such as edge monitoring and Kalman filtering. The field implementation results showed that this real-time tracking algorithm achieves 98% accuracy. For the burial of fiber-optic cables, Vidovic et al. proposed the use of existing fiber-optic cables near tracks for monitoring, which could greatly reduce monitoring costs [53]. However, the key point is to accurately locate the buried positions, especially those of the winding parts and redundant parts of cables. In addition, “tap tests” must be performed to obtain the corresponding relationships between the spatial positions of fiber-optic cables and channel numbers, which take considerable time to process. Vidovic et al. suggested the use of train platforms, bridges, and other positions as reference points in engineering practice [53].

#### 3.1.2. Rail Track Health Monitoring

Ever-increasing railway traffic not only drives social and economic development but also aggravates the wear and tear of tracks. At present, rail track health monitoring mostly relies on manual inspections and conventional deformation monitoring devices, such as track inspection vehicles, accelerometers, and strain gauges near the tracks. However, both manual monitoring and track inspection vehicle monitoring have certain monitoring periods and it is impossible to evaluate the health status of tracks in real time. Some sensors, such as accelerometers and strain gauges, cannot be deployed around tracks in a large-scale and intensive manner for monitoring due to their high prices.

Some researchers have proposed the use of distributed fiber-optic sensing (DFOS) technologies to evaluate the health status of tracks, such as Brillouin optical time domain analysis (BOTDA) [54] and optical frequency domain reflectometry (OFDR) [55]. These sensing technologies have a high sampling density and can obtain huge amounts of information on the health status of tracks. However, BOTDA cannot realize real-time sensing and OFDR has limitations in detection distance. On the contrary, DAS has the potential to greatly extend detection distances with super-high sensitivity, which could provide an ideal solution to track health monitoring.

**Figure 5:**
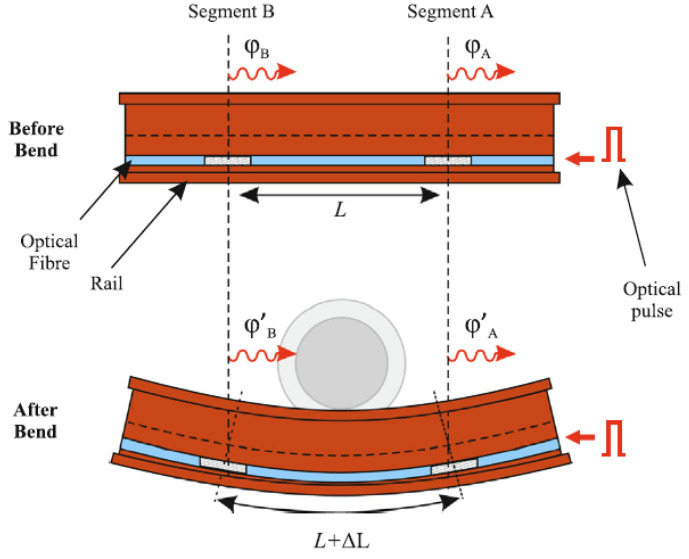
Schematic diagram of fiber-optic cable layout and sensing. Reprinted with permission from Ref. [35]. 2020, Elsevier. In the figure, *φ* represents the phase information, *L* is the length, and Δ*L* is the change in length.

In recent years, many successful trials have been introduced [49,50,51]. Milne et al. pointed out that DAS can be used to measure track deflections and sleeper end loads [35]. They pasted sensing cables under rails and performed back calculations using the track strain data that were recorded by the sensing cables, as shown in Figure 5. To verify the feasibility of this measurement method, the results from the DAS, strain gauges, and digital image correlation (DIC) were compared, which showed good consistency. Guo et al. developed an intelligent detection method for track slab deformation based on a random forest model and carried out field experiments to verify it [56]. The test results showed that this intelligent algorithm can effectively identify deformations in track slabs and the recognition rate reaches 96.09%. Wang et al. also pointed out that DAS has the potential to detect track health status [57]. They used deep convolutional networks to detect the health status of tracks and conducted field experiments to verify them. The test results showed that the accuracy of the recognition method reaches 98.04%. The above experimental results demonstrate the application potential of DAS in the field of track health monitoring. However, these applications also face some challenges. On the one hand, some trace vibration characteristics that could identify issues may be submerged within strong external noise. On the other hand, analyzing the health status of tracks based on artificial intelligence technology requires a large amount of labeled data, which is difficult to obtain [57]. In the future, researchers should work closely with railway departments to develop intelligent algorithms with stronger denoising and higher recognition capabilities. In addition, researchers could also combine DAS with other technologies to jointly monitor the health of railway tracks in order to compensate for the lower detection ability of a single detection technology, thereby breaking through the abovementioned developmental bottlenecks.

#### 3.1.3. Roadbed Velocity Structure Imaging

When trains are running at high speeds, some of their energy is transmitted in the form of seismic waves due to the extrusion, friction, and collision between the trains and the tracks. The seismic waves excited by trains are good active source seismic wave fields, which contain the structural and dynamic characteristics of the tracks and roadbeds [58,59,60]. By analyzing changes in seismic waves, structural defects in roadbeds can be monitored and disaster warnings for high-speed railways can be realized.

Restricted by multiple factors, such as cost, power, and burial location, it is difficult for traditional seismic sensing devices (nodal seismographs, narrowband geophones, broadband seismographs, etc.) to be deployed on a large scale and for long periods of time along railways. DAS breaks through the above limitations nicely. The detection units on fiber-optic cables do not need to be powered separately, only the interrogation units need to be supplied with power. In addition, there is a large number of existing communication fiber-optic cables along railways. As long as the cables are connected to interrogation units, we can form long-distance, real-time and dense seismic sensing networks. Recently, researchers have monitored and analyzed seismic signals generated by moving trains with the aid of DAS [61,62]. Shao et al. extracted surface wave signals from DAS records through seismic interference technology and then used the multi-analysis surface wave (MASW) method to carry out inversion work. Finally, they successfully obtained the shallow shear wave velocity structure under a roadbed, which fully demonstrates the feasibility of DAS in roadbed velocity structure imaging [61].

However, research in this field is still in its infancy. On the one hand, trains are moving sources and the seismic waves they excite are very complex. On the other hand, DAS is only sensitive to seismic waves that propagate in specific directions due to its direction sensitivity. The above factors limit the further development of DAS in the field. It is believed that with the continuous deepening of research, these bottlenecks can be broken through and DAS can be integrated with high-speed rail seismology in the future.

### 3.2. Highway Traffic Monitoring

Highway traffic is an essential part of people’s lives. To monitor highway traffic, a variety of fixed (radar guns, road sensors, cameras, etc.) and mobile (vehicle GPS, mobile phones, etc.) monitoring technologies have been developed in recent years. The former can provide high-resolution monitoring data but their installation and maintenance costs are high and their space coverage is relatively low. The latter have high space coverage but their data collection frequency is low, so they cannot perform real-time monitoring and may also involve personal privacy problems [63]. Therefore, new traffic monitoring systems are urgently required.

**Figure 6:**
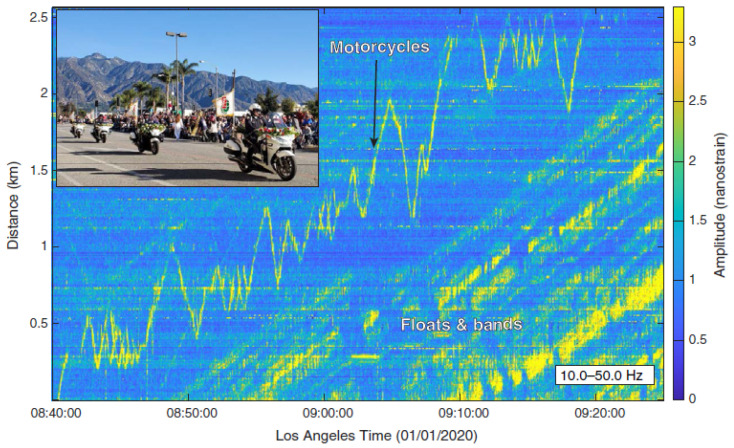
Seismic records (10.0–50.0 Hz) showing the vibrations caused by motorcycles, floats, and bands. Reprinted with permission from Ref. [67]. 2020, Seismological Society of America.

**Figure 7:**
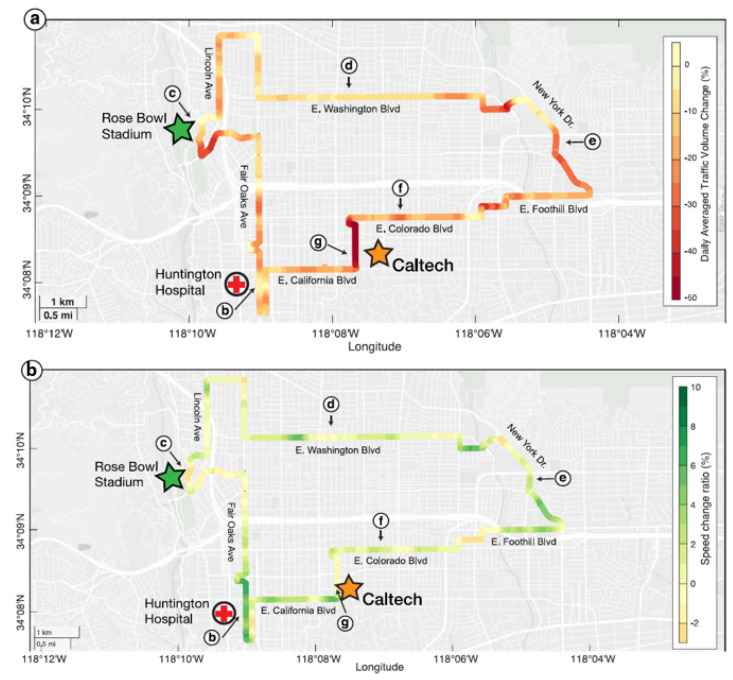
(**a**) A map showing the changes in average daily traffic volume before and after lockdown using DAS-based transportation analysis; (**b**) a map showing the degree of change in mean traffic speed before and after lockdown using DAS-based transportation analysis [63].

DAS systems could provide an alternative for highway traffic monitoring. Because fiber-optic cables are buried under roads, this detection method has strong concealment qualities and the cables embedded underground can avoid physical damage during long-term monitoring. In recent years, there have been several good examples of applying DAS to highway traffic monitoring [64,65,66]. Wang et al. monitored the Pasadena Rose Parade using a communication cable under the road [67]. By analyzing the vibration information collected by the cable, they successfully identified traffic characteristic signals, such as pedestrians, motorcycles, and floats (Figure 6). They also proposed a method to measure road traffic flow and speed and analyzed urban road traffic conditions before and after the outbreak of COVID-19 [63]. The results are shown in Figure 7. It can be clearly seen that the overall traffic flow in the city decreased after the outbreak of COVID-19, while the speed increased. They also compared the monitoring results from DAS to those from other monitoring technologies and found that they had good consistency, which successfully proves the feasibility of DAS in highway traffic monitoring. Catalano et al. proposed the application of the Hough transform to vehicle counting and demonstrated an algorithm for the automatic detection and counting of vehicles [68]. Field tests showed that the accuracy rate of the algorithm reaches 73%.

Although DAS has great application potential in highway traffic monitoring, it also faces many challenges. For instance, the deformation coupling of fiber-optic cables in some highway sections is limited and it is impossible to accurately record the vibration signals generated by traveling vehicles as during heavy traffic congestion periods, it is difficult to accurately distinguish the vibration signals generated by each type of vehicle, which would adversely affect the traffic flow statistics.

### 3.3. Pipeline Safety Monitoring

As safe and cheap transportation devices, pipelines are widely used for the transportation of oil, natural gas, and other products. The real-time monitoring of pipeline operation status can effectively maintain pipeline safety and prolong pipeline service life. DFOS is a technology that developed rapidly in the 1980s. It has been widely used in pipeline safety monitoring in recent years. For instance, distributed temperature sensing (DTS) technology is used to monitor pipeline leakages [69,70] and distributed strain sensing (DSS) technology is used to monitor pipeline deformations [71,72,73]. However, they are static monitoring technologies and cannot perform the real-time and dynamic monitoring of pipelines. The vigorous development of DAS has provided new ideas and solutions for the dynamic monitoring of pipelines.

#### 3.3.1. Pipeline Intrusion Detection

**Figure 8:**
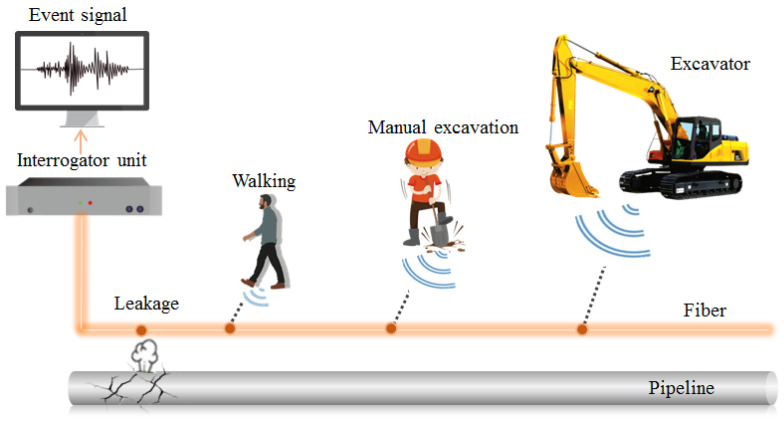
Schematic diagram of pipeline intrusion and leakage monitoring based on a DAS system.

Intrusion detection is another important function of DAS. By acquiring and analyzing abnormal vibration signals from cables around pipelines, the location and type of intrusion events can be roughly determined, as shown in Figure 8. In 2009, Tanimola et al. simulated several common intrusion events around a pipeline and summarized the characteristics of the vibration signals from the cable [74]. In the same year, European researchers also successfully applied DAS to detect oil theft incidents. These cases fully demonstrate the great potential of DAS in pipeline intrusion monitoring and thereafter, an increasing number of field monitoring investigations have been carried out [75,76,77,78].

Some researchers have proposed the combination of DAS with artificial intelligence algorithms, such as pattern recognition, artificial neural networks, and support vector machines, to process huge amounts of real-time monitoring data [79,80,81,82]. Recent works have used a variety of pattern recognition algorithms to classify and identify different types of mechanical intrusion signals, e.g., large excavators hitting the ground, large excavators scraping the ground, and small excavators moving along the ground [83,84,85]. These algorithms have shown good classification and recognition effects and can significantly reduce false alarm rates in intrusion detection. To solve the difficulty in the classification of human and animal activities in complex and harsh monitoring environments, He et al. designed a dual-stage recognition network [79]. They carried out field experiments and explored the recognition accuracy of this network for five different types of intrusion events, such as animal intrusion, human intrusion, and mechanical excavation. The results showed that the average recognition rate reaches 97.04%. Furthermore, Yang et al. proposed a semi-supervised learning method for long-distance pipeline intrusion monitoring, which effectively improves our ability to identify and locate intrusion events under low signal-to-noise ratio conditions [80]. Based on DAS and pattern recognition systems (PRS), a review of the literature that introduces several machine learning techniques for pipeline surveillance systems can be found in [86].

#### 3.3.2. Pipeline Leakage Monitoring

Negative pressure waves or noise are generated at the positions of leakages in pipelines and these abnormal vibration signals can be recorded by the surrounding fiber-optic cables. Therefore, by analyzing the real-time data recorded by DAS, the locations of pipeline leakages can be found and changes in internal pressure can be inferred. According to installation positions of fiber-optic cables, the monitoring methods can be divided into two types: one method type directly pastes cables onto pipe walls and the other buries cables in the ground soil near pipelines. The former can receive strong signals but the composition of the signals is complex, which means that data analysis algorithms are difficult to implement. In addition, pasting fiber-optic cables is also time-consuming. The latter is easier to implement in engineering practice, especially for buried pipelines. However, there should be suitable distances between the fiber-optic cables and the pipelines and the signal strength received by the cables is relatively weak, which may affect the monitoring sensitivity of DAS to small leakage events.

In recent years, many successful trials have been performed [87,88,89,90]. In 2015, Wu et al. applied DAS to pipeline leakage monitoring. They investigated the response of surface-glued fiber-optic cables by changing the internal pressure and the diameter of the leak hole [91]. The test results showed that DAS can respond well when the leak diameter is greater than or equal to 4 mm and the internal pressure is higher than 0.2 MPa. To explore whether DAS has the ability to monitor small leakage events, Stajanca et al. conducted laboratory experiments [34]. They wound a sensing cable helically around a pipeline and simulated a pipeline leakage event with a low leakage rate. By analyzing the time domain and frequency domain features of the recorded data, they successfully detected and located the leak event, thereby proving that DAS is fit for monitoring small pipeline leakage events (i.e., leak rates down to 0.1% of the pipeline flow volume). In an experiment carried out by Zuo et al., fiber-optic cables were placed outside of a pipeline [33]. Based on the wavelet transform and empirical mode decomposition detection algorithms, they significantly improved the signal-to-noise ratio of monitoring signals (the signal-to-noise ratio increased to 18.28) and used the frequency domain cumulative average algorithm to accurately locate pipeline leakages, thereby proving that DAS can realize the non-contact monitoring of pipeline leakages.

However, the above experiments were carried out indoors. Considering the complexity of field environments, the performance of DAS may be affected. For instance, when pipeline leakages are in the early stages, the abnormal vibration signals are easily submerged by external noise, resulting in leakage events being underreporting. Additionally, when external interference is too strong, false alarms are easily caused. To improve the leakage monitoring capability of DAS, Wang et al. proposed the combination of DAS, DTS, and machine learning algorithms to monitor pipeline leakages [87]. Their experimental results showed that this method can effectively reduce false positives for leakage events. The average leakage event recognition rate reaches 98.57%. Furthermore, the recognition time of this method is only 6.79 ms. It is believed that this method will play an increasingly important role in monitoring pipeline leakages in the future.

### 3.4. Tunnel Structure Health Monitoring

As essential public transportation facilities, tunnels can greatly shorten driving distances, save transportation costs, and protect ecological environments. However, in the process of tunnel construction, geological disasters, such as water leakages and floods, often occur [92]. These incidents pose a serious threat to the health of tunnels. Therefore, there is an urgent need for real-time and comprehensive evaluation technologies to monitor tunnel health status.

DAS provides a brand new tool for tunnel health monitoring [93,94,95]. Based on a scattering-enhanced fiber-optic DAS system, Hu et al. performed the intelligent monitoring of a tunnel ring [94]. They laid cables on a steel ring structure to monitor vibration frequency. Using the monitoring data, they assessed and classified the health status of the steel ring based on machine learning algorithms. The recognition accuracy of the final test was as high as 97.8%, indicating that DAS can be used to monitor the health condition of tunnel structures.

Aiming to mitigate potential disaster events during tunnel construction, Zhang et al. proposed a DAS-based disturbance event identification method [95]. They laid cables along a tunnel lining and used a random forest algorithm to analyze the DAS data. They successfully identified various vibration events during tunnel construction, including unexpected disasters, such as rockfalls. Its recognition accuracy rate is as high as 92.31%.

### 3.5. Border Security Monitoring

**Figure 9:**
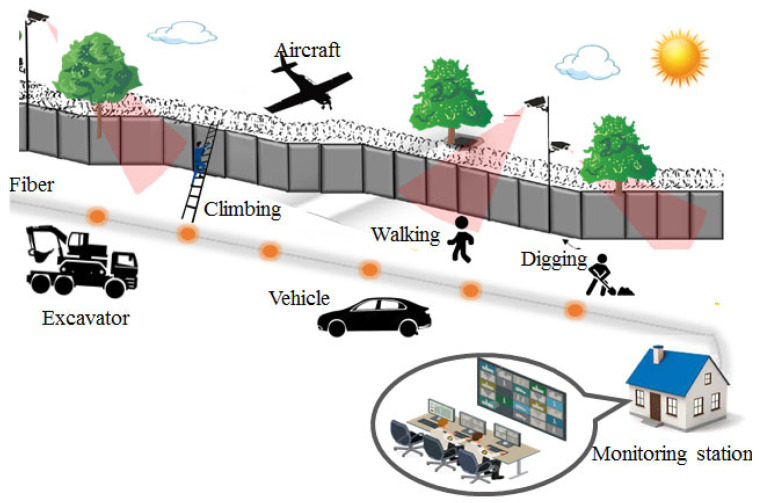
Smart border security system. Reprinted with permission from Ref. [97]. 2020, SCITEPRESS.

In this era of cross-border terrorism and illegal immigration, many countries around the world are actively seeking ways to effectively protect their borders. Traditional border security technologies (radars, electro-optical/infrared, fencing, etc.) have shortcomings, such as being susceptible to electromagnetic and terrain interference and requiring the frequent maintenance of detection devices. DAS offers a new approach to border security. It can perform long-distance detection, classification, and localization, which enables timely responses to various border intrusion events (as shown in Figure 9). In addition, DAS provides low-noise and high-spatial resolution acoustic and seismic sensing that can detect and identify personnel, vehicle, and even aircraft intrusions. Therefore, DAS-based border security technology is regarded as a future development direction in the border security field.

Duckworth et al. investigated DAS responses to different intrusion events and found that DAS can not only capture the vibration signals of personnel footsteps but also respond well to intrusion events that are difficult to detect using conventional security technologies, such as digging, gunshots, underground excavation, and aircraft intrusion [96]. This fully demonstrates the application potential of DAS in border intrusion monitoring. However, there are some challenges; for instance, how to quickly discover and locate intrusion events within huge amounts of monitoring data and how to eliminate the interference of non-human invasion events (such as animal activities). To solve the above challenges, on the one hand, artificial intelligence technology (machine learning, deep learning, etc.) could be used to improve the efficiency of data processing or on the other hand, comprehensive analysis platforms for multivariate data (DAS data, radar data, video surveillance data, etc.) could be developed to analyze multivariate information and make comprehensive decisions in order to reduce the false positive and false negative rates of intrusion events.

### 3.6. Other Applications

In addition to the applications listed above, there are some other cases that use DAS to monitor linear infrastructures. For instance, cables buried in airport runways can be used for aircraft monitoring [98]. A pioneering work was carried out by the scientific research team of Beijing Jiaotong University. They presented an aircraft acoustic signal detection system based on DAS and analyzed the seismic waves excited by aircrafts. The 5 Hz region was shown to be the most meaningful frequency range. Researchers have also focused on the health monitoring of wind turbine towers and proposed methods to detect the structural phenomena associated with loose bolts and material damage within the towers [99]. Based on the amplitude, frequency, power spectrum, and other characteristics of the data, loose bolts and material damage within towers can be monitored, which is of great significance for the maintenance of wind towers.

## 4. Challenges and Future Trends

### 4.1. Challenges

Various studies have proven the feasibility and reliability of DAS for linear infrastructure monitoring. However, DAS is not universally effective and has some shortcomings compared to other fiber-optic sensing technologies. Table 3 compares the advantages and disadvantages of several fiber-optic sensing technologies. In general, the current challenges faced by DAS can be subdivided into two categories: technical challenges and challenges in applying DAS to engineering practice. The technical challenges of DAS are as follows:

- Directional sensitivity. Compared to three-component vibration detection devices (seismometers, accelerometers, etc.), DAS only has sensitivity along the axial direction of fiber-optic cables. DAS is highly sensitive to longitudinal waves propagating along fibers and to transverse waves propagating at 45° to fibers. It is only weakly sensitive to broadside waves [9]. In addition, when seismic wavelengths are close to the gauge length, the directional sensitivity of DAS becomes more complex [103,104];
- Complex amplitude responses. The absolute amplitude information from DAS signals is essential to amplitude-based studies, such as attenuation analysis, source inversion, and subsurface imaging [17]. However, DAS amplitude responses are complex. Previous studies have shown that the factors affecting DAS amplitude responses include gauge length [94], cable structure (e.g., tight-buffered versus loose-tube) [29], field deployment method (e.g., direct burial versus conduit embedding) [105], initial strain state [106], and near-surface geological conditions [107]. These complex amplitude responses cause trouble for practitioners when analyzing and interpreting data, which limits the further application of DAS to a certain extent;
- Sensing distance and spatial resolution. The sensing distance and spatial resolution of DAS are closely related to pulse width. The shorter the pulse width, the higher the spatial resolution but the shorter the sensing distance. That means that there is a contradiction between the sensing distance and the spatial resolution. For linear infrastructure monitoring (such as crack detection in railway tracks, micro-crack detection in large infrastructures, border intrusion location, etc.), we expect DAS to have a higher spatial resolution (at the cm level) and longer sensing distances (hundreds of km). Recently, researchers have carried out numerous investigations into overcoming this challenge. For instance, Lu et al. achieved a spatial resolution of 30 cm, a sensing distance of 19.8 km, and a vibration sensing signal-to-noise ratio of 10 dB using the optic swept pulse method [108].

The main challenges of applying DAS to engineering practice are as follows:

- Spatial positioning of fiber-optic cables. The spatial position of each sensing channel of fiber-optic cables is a piece of important information that investigators must consider when analyzing DAS data. Investigators need to perform tap tests to obtain the corresponding relationships between the spatial positions of fiber-optic cables and sensing channels. For cases that need to deploy fiber-optic cables on site, investigators can accurately obtain the spatial locations of fiber-optic cables (especially the locations of redundant sections). However, when using existing fiber-optic cables for monitoring (such as communication fiber-optic cables near railway tracks and underground communication fiber-optic cables under urban roads), it is very difficult to obtain the accurate spatial location information of the fiber-optic cables. Because investigators cannot obtain detailed information about the layout conditions of existing fiber-optic cables (the layout of fiber-optic cables is not always in ideal straight lines, there can be many redundant cables, and the cables may twist at some positions), they can only verify the information through a large number of tap tests to obtain the general spatial layout of the fiber-optic cables, which is very time-consuming and laborious;
- Deformation coupling. For linear infrastructures with large spans, it is a challenge to ensure that fiber-optic cables always maintain valid coupling conditions with the engineering structures or the ground. When the coupling between fiber-optic cables and their surroundings is weak, the transmission effects of strain and vibrations are greatly affected, thereby decreasing the signal-to-noise ratio of the recorded data. Many field tests have fully proven this point [109,110]. In recent years, many researchers have carried out a lot of research works on cable–soil deformation coupling. The results of laboratory experiments have demonstrated that the structures of fiber-optic cables affect the interaction and strain transfer between fiber-optic cables and soil, thereby affecting the quality of the monitoring data [40,111]. Although a lot of valuable works on cable–soil deformation coupling have been carried out, in view of the complex coupling mechanism and the changeable application environments during vibrations, deformation coupling between fiber-optic cables and their surroundings needs to be further explored in the future;
- Data storage, transmission, and processing. Since each sensing unit on cables collects information at a high frequency, the records are very large. The amount of information collected by tens of kilometers of fiber-optic cables in a day can even reach the TB level. These massive amounts of data make storage, transmission, and processing complex and time-consuming tasks. In terms of data storage, some DAS manufacturers provide filtering and compression systems that can reduce the number of records. However, some valuable records can be lost after compression. In terms of transmission, few wireless network platforms support the transmission of DAS records, so records are generally transmitted through hard disks and other methods. In terms of processing, although artificial intelligence algorithms can improve processing speeds, in the face of TB-level amounts of monitoring data, the speed of data processing still needs to be improved. In addition, jointly analyzing DAS data and other monitoring data (DTS data, geophone data, etc.) is also a big challenge.

### 4.2. Future Trends

Although DAS faces many challenges, as mentioned above, its application prospects are broad. In linear infrastructure monitoring, the following five trends will be realized in DAS-based linear infrastructure monitoring in the future:

- Improvements in the performance of DAS systems (sensitivity, spatial resolution, sensing distance, frequency response range, etc.). In order to expand the application potential of DAS and improve its monitoring capabilities in complex and harsh environments, it is necessary to improve the monitoring performance of DAS systems. For instance, improving the spatial resolution (cm level) could not only increase the equivalent sensing channel of DAS but also expand the maximum strain/vibration range [112]. Expanding the frequency response range (MHz level) could make DAS applicable in the field of the nondestructive detection of engineering structures [113]. Increasing the sensing distance (hundreds of km) could provide DAS with more advantages in the fields of pipeline, railway, and border monitoring. In recent years, investigators have carried out many studies on this topic. For instance, in order to improve the sensitivity of DAS, investigators have proposed fading suppression [114] and laser phase-noise compensation techniques [115,116], as shown in Table 4. These techniques allow DAS the sensitivity to detect nano-strains. Investigators have also proposed that the sensitivity of the DAS could be increased (up to 2.2 times) by building coils inside sensing cables, which has been verified by field experiments [117]. In addition, the design of special fiber-optic cables is also regarded as an essential measure to improve the detection capability of DAS. Table 5 shows several specially designed fiber-optic cables and their performance. In terms of sensing distance, as far as we know, the longest distance is 175 km. It is believed that with the continued deepening of research in the future, the monitoring performance of DAS will be further improved;

**Table 4:** Comparison of various fiber-optic sensing techniques [118].

Year	Method/Technique	Sensitivity	Reference
2018	Chirped pulse Phi-OTDR with phase-noise compensation	$5p\varepsilon /\sqrt{Hz}@1\;kHz$	[115]
2019	Pulse compression with phase-noise compensation	$92.84p\varepsilon /\sqrt{Hz}@500–2500\;Hz$	[116]

**Table 5:** Performance summary of CSE and DSE fibers [93].

	Fabrication Method	SNR Enhancement (dB)	Sensing Distance (km)
CSE fibers	Continuously inscribe Bragg gratings	15	1
	Highly doped fibers	14	1.9
DSE fibers	UV exposure	5.5–21.1	50
	Femtosecond laser inscription	13–15.8	9.8
- Breakthrough of the directional sensitivity limit. In response to the directional sensitivity of DAS, many research teams have proposed designs for the structures of fiber-optic cables (such as spirally winding fiber-optic cables) to meet the needs of multi-component measurements [119,120,121,122]. For instance, Hornman et al. designed a helically wound fiber-optic cable. They spirally wound the cable at a certain angle to obtain vibration signals at different angles. They found that when the cable is wound at an angle of 30°, the cable almost has the same sensitivity to vibrational waves in all directions [121]. On this basis, Lim and Save proposed an acquisition system using five equally spaced helical fibers and a straight fiber to obtain six different strain projections, which reconstructs all components of 3D strain tensors at any location along fiber-optic cables [122]. They verified the feasibility of the method through numerical simulations. In addition, optimizing the geometric layout of fiber-optic cables is also regarded as a measure that could improve the directional sensitivity of DAS. By designing alternative geometric layouts for fiber-optic cables (such as umbrella and checkerboard layouts) [123], the vibration signals from multiple directions can be captured to obtain more comprehensive vibration information and improve the directional sensitivity of DAS;
- Development of data processing software and risk assessment systems. In recent years, a series of computational intelligence technologies, including fuzzy logic, genetic algorithms, wavelet analysis, machine learning, and deep learning, have developed rapidly. These computing algorithms provide the possibility for the efficient and diversified processing of DAS data. At the same time, these intelligent technologies can also help researchers and practitioners to mine more potential information, thereby helping researchers and practitioners to conduct scientific research. The establishment of risk assessment systems will help management departments to grasp the health status of linear infrastructures in a timely manner. If key detected parameters exceed the predetermined thresholds, the risk assessment systems will promptly notify the management departments through SMS, email, etc., and the management departments can take corresponding emergency measures to avoid major personnel and property losses;
- Establishment of a large-capacity network data sharing platform. The establishment of a data sharing platform could not only effectively relieve the pressure of data transmission but also allow more researchers and practitioners to conduct scientific explorations using shared DAS data, thereby promoting the development of DAS;
- Preparation of guidelines to improve standardization in field monitoring. With the increasing number of engineering practices, there is an urgent need for countries and regions to develop relevant standards, norms, and guidelines to implement monitoring. In recent years, experts and scholars from all over the world have successively published several guides for the field of DFOS. However, specifications and guides for the application of DAS in the field of linear infrastructure monitoring are still lacking and need to be complied by experts and scholars to enable standardized engineering monitoring;
- Breakthrough the technical bottleneck for interrogation units. Although sensing cables are cheap, interrogation units are expensive. The price of a DAS interrogation unit is nearly RMB one million, which limits the application and promotion of DAS to a certain extent. We urgently need to break through the technical difficulties and reduce the production costs of equipment, thereby allowing the large-scale promotion and application of DAS.

## 5. Conclusions

As a new type of fiber-optic sensing technology for long-distance, distributed, and real-time acoustic monitoring, DAS has received extensive attention from researchers and practitioners since its inception. With the continuous development of DAS, its application scenarios are constantly expanding and adapting. For linear infrastructure monitoring, a large number of field investigations have been conducted. This paper presented a review of research and development activities in linear infrastructure monitoring based on DAS. The sensing principles and performance of DAS were briefly described and the different installation and layout methods for fiber-optic cables were summarized. Compared to ordinary fiber-optic sensing technologies, DAS can greatly expand the detection distance (over 100 km), shorten the sensing response time (real-time responses), and also offer a very high spatial detection density (detection spacing reaching the cm level), which can enable the monitoring of the health conditions of linear infrastructures.

Although the application of DAS to linear infrastructure monitoring faces many challenges, such as the huge amounts of monitoring data, the directional sensitivity of fiber-optic cables, and the difficulties in processing data, the application potential of DAS is broad. It is believed that with improvements in DAS sensing performance, the research and development of new cables, breakthroughs in multi-component sensing technology, and the development of data processing software, DAS will play an increasingly important role in linear infrastructure monitoring in the future.

## Figures and Tables

**Table 1:** Comparison of the basic parameters of DAS interrogators.

Parameter	HDAS (Aragon Photonics)	Helios DAS (Fotech)	MS-DAS2000 (Ovlink)	IDAS3 (Silixa)	CRI-4400 (Halliburton)	QuantX (OptaSense)
Strain sensitivity (*ε*)	10^−9^	10^−9^	10^−9^	10^−9^	10^−9^	10^−9^
Spatial resolution (m)	10	2	2	1	1	2
Sensing range without repeaters (km)	70	50	20	50	50	50

**Table 2:** Different fiber-optic cable installation and layout methods and their characteristics.

Method	Measurement Objects	Advantages	Disadvantages
Fixture-fixed installation [43]	Tunnels, pipelines, etc.	Easy installation and low cost	Poor coupling at some positions
Slotted and glued installation [44]	Formed reinforced concrete structures	Good overall coupling effect	Time-consuming
Spot welding installation [45]	Steel beams, rails, and other metal structures	Easy installation and low cost	Poor coupling at some positions
Groove installation [46]	Geotechnical structures	Strong concealment and good overall coupling effect	Time-consuming

**Table 3:** Comparison of various fiber-optic sensing techniques [39].

Technique	Specifications	Measurement Parameters	Characteristics	Limitations
FBG	Type: quasi-distributed Range: ≈100 channels [100] Spatial resolution: 2 mm	Temperature, strain, pressure, and displacement	Simple structure, small size, lightweight, good compatibility, low optical loss, and high sensitivity	The grating subsides under high temperatures and chirps easily under sticking and compression; it is easily damaged when processed and some information is blocked because of the quasi-distribution
DAS	Type: distributed Typical sensing range: 1–50 km Typical spatial resolution: 5–10 m	Strain, Temperature, vibrations, sound waves, and seismic waves	Single-end measurement, wide response bandwidth, large measuring range, and dynamic monitoring	Huge amounts of monitoring data; directional sensitivity
OFDR	Type: distributed Typical Sensing Range: 1–50 m Typical Spatial Resolutions: 1–2 cm	Strain and temperature	High sensitivity, High S/N ratio, and suitable for static measurements	Not suitable for long-distance monitoring; nonlinearity effects [101]; laser intensity noise [102]
BOTDA	Type: distributed Typical sensing range: 1–50 km Typical spatial resolution: 1–10 m	Temperature, displacement, deformations, And deflections	Double-end measurement, large measuring range, and high accuracy for the measurement of absolute temperature and strain values	Unable to detect breakpoints; high monitoring risks brought by double-end measurement

## Data Availability

Not applicable.
